# Knowledge of Depression and Malingering: An Exploratory Investigation

**DOI:** 10.5964/ejop.v16i1.1730

**Published:** 2020-03-03

**Authors:** Ashley Cartwright, Rebecca Donkin

**Affiliations:** a Behavioural Sciences, School of Human and Health Sciences, University of Huddersfield, Huddersfield, United Kingdom; b Department of Psychology, Leeds Trinity University, Leeds, United Kingdom; Glasgow Caledonian University, Glasgow, United Kingdom

**Keywords:** malingering, mental health literacy, depression, deception, cognitive load

## Abstract

Malingering mental disorder for financial compensation can offer substantial rewards to those willing to do so. A recent review of UK medico-legal experts’ practices for detecting claimants evidenced that they are not well equipped to detect those that do. This is not surprising, considering that very little is known regarding why individuals opt to malinger. A potential construct which may influence an individual’s choice to malinger is their knowledge of the disorder, and when one considers the high levels of depression literacy within the UK, it is imperative that this hypothesis is investigated. A brief depression knowledge scale was devised and administered to undergraduate students (N = 155) alongside a series of questions exploring how likely participants were to malinger in both workplace stress and claiming for benefit vignettes. Depression knowledge did not affect the likelihood of engaging in any malingering strategy in either the workplace stress vignettes or the benefit claimant vignettes. Differences were found between the two vignettes providing evidence for the context-specific nature of malingering, and an individual’s previous mental disorder was also influential.

The worldwide prevalence of depression is increasing (Global Burden of Disease study, 2015) and it is estimated that depression accounts for half of all psychiatric consultations and 12% of all hospital admissions (Kuo, Tran, Shah, & Matorin, 2015). Estimations for the global prevalence of depressive disorders is estimated at 3.5% in males and 5.1% in females (World Health Organisation, 2017) and the most debilitating depressive disorder, Major Depressive Disorder (MDD) is estimated to affect 7% of society according to the American Psychiatric Association (APA, 2013). This highly prevalent mental disorder seriously reduces the quality of life and also produces serious economic and social implications (Monaro, Gamberini et al., 2018). Such economic implications arise through a variety of avenues including the cost of treatment, the cost for employers covering absences from work, and the cost associated with compensation. Indeed, the economic costs associated with depressive disorders are high and these are surely exacerbated by those willing to feign or exaggerate symptomology.

In the United Kingdom, research has demonstrated that residents’ do not perceive fraudulently claiming mental disorder for financial compensation to be a severe act of criminality (Cartwright & Roach, 2016). This behaviour is defined clinically within the DSM-5 as malingering (APA, 2013). Estimations of the prevalence of malingered mental disorders vary substantially depending upon the context but within contexts involving compensation, depressive disorders are suggested to be malingered within 16.08% of cases (Mittenberg, Patton, Canyock, & Condit, 2002). One situation which presents itself to those willing to commit this type of behaviour is the road traffic accident (RTA). Research has alluded to fraud being rife within UK RTA claims through figures illustrating that between 2006-2011 the number of RTAs reduced by 20%, whilst the number of personal injury claims because of such accidents increased by 60% (Merten et al., 2013). A similar paradox to the one outlined within RTA claims is also evident within UK disability claims through a 44% increase in claims whilst the average health status according to the Department for Health and Pensions continues to improve (Merten et al., 2013).

Alongside fraudulent claims occurring within benefit payments and RTA claims, a further category of claims which are vulnerable to malingered depression, are psychological injury claims in the workplace. Yoxall, Bahr, and O’Neill (2017) suggest that in Australia between 9-31% of workplace compensation cases for psychological injury involve some form of symptom exaggeration. Cartwright and Roach (2016) provide further insight into this issue in the UK and suggest that only 6% of individuals questioned would be likely to make up symptoms of mental disorder after having a traumatic experience in the workplace. The claims culture in the UK is certainly a concern and the economic ramifications of fraudulent claims are vast affecting the regular insurance/ taxpayer, the insurance industry, and the government. Therefore, it is important to gain an in-depth understanding of those who commit fraudulent claims for psychological and physical injury.

The first theoretical model of malingering labelled the criminological model was proposed by Rogers (1990) and this explanation is adopted in the DSM-5 (APA, 2013), whereby malingerers are regarded as anti-social and bad. However, scholars are yet to find a definitive link between psychopathy or antisocial personality disorder and malingering (Wooley, 2013). A differing theoretical approach suggested by Rogers (1990) is the pathogenic model, which argues that individuals malinger due to an underlying psychological reason. The model posits that the production of malingered symptomology is an attempt to control underlying psychopathology (Rogers, 1990). This model of malingering is not well supported (Rogers, 2008) and is better placed to explain factious disorder. The final theoretical model is the adaptation model which suggests that malingering is due to the context of the situation and the individual making the best of a difficult situation through the consideration of rational choice (Rogers, 1990).

At present, there is no clear and accepted explanation for why individuals malinger, although research has indicated that in a sample of Australian Psychologists the adaptational model receives the most support (Yoxall, Bahr, & Barling, 2010). Other than the models suggested by Rogers (1990) very few attempts have been made to explain the behaviour. Typically, research has focused upon examining the response styles of malingering within one context (Peace & Masliuk, 2011), however, should one accept the premise of the adaptational model, then understanding the contexts in which an individual malingers is crucial. Research has demonstrated that feigning symptoms which have arisen because of a sexual assault, produce more exaggerated symptoms than RTA respondents (Edens, Otto, & Dwyer, 1998). Furthermore, Peace and Masliuk (2011) reported that where the following motives, no motivation, compensation motive, attention-seeking motive, and revenge motive were implicit this resulted in different extents of symptom exaggeration. Participants in the revenge and compensation vignettes obtained the highest self-report symptomology scores (Peace & Masliuk, 2011).

The definition given by the DSM-5 describes malingering as a singular construct despite research illustrating that malingering occurs at different levels (Walters et al., 2008). Resnick’s (1977) proposes three types of malingering that can occur and these are as follows, pure malingering (Pure-M), partial malingering (Par-M), and false imputation (F-Imp). Pure-M refers to a claimant who experiences no symptoms of disorder or impairment despite claiming to suffer symptoms. Par-M refers to claimants who do suffer symptoms of disorder or impairment but exaggerate their symptoms to attain higher rewards. Lastly, F-Imp refers to claimants who experience genuine symptoms of disorder or impairment but attribute these to an event which did not cause the symptoms.

Research indicates that Par-M is viewed as the least severe followed by F-Imp, and then Pure-M (Cartwright & Roach, 2016). Such findings are particularly important due to Par-M and F-Imp being argued to be the most difficult to detect due to the claimant experiencing some genuine aspect of the disorder (Cartwright, Roach, Wood, & Wood, 2016; Hall & Hall, 2006; Resnick & Knoll, 2005). Cartwright, Roach, and Armitage (2019) examined the assessment methods used within the UK to detect malingered mental disorder and uncovered numerous issues within clinicians’ assessments in compensation cases. As a result, a sister article was produced providing guidance on the most appropriate assessment tools to employ for detecting malingered psychopathology (Cartwright, 2019). Notably, it should be acknowledged that an extensive review of the methods for detecting malingering is not attempted here but should the reader require this Rogers’ (2008) seminal text is advised.

Extensive research within the deception literature indicates that telling a lie is cognitively taxing (e.g. Monaro, Gamberini, & Sartori, 2017; Monaro, Galante et al., 2018; Wang, Spezio, & Camerer, 2010; Vrij, 2008; Zuckerman, DePaulo, & Rosenthal, 1981). Through this knowledge, that lying is cognitively taxing, has recently led to the development of behavioural methodologies to detect malingering (Ferrara et al., 2016; Monaro, Galante et al., 2018; Sartori, Agosta, & Gnoato, 2007; Sartori, Orrù, & Zangrossi 2016). Such methods of lie detection are possible by measuring behavioural reactions such as response time which may be indicative of cognitive load. Such methods have demonstrated impressive discrimination rates when differentiating malingers from genuine respondents in a variety of conditions including: whiplash (Sartori et al., 2007), phantom limb pain (Ferrara et al., 2016), psychogenic amnesia (Sartori et al., 2016), and more recently depression (Monaro, Toncini et al., 2018).

In Monaro, Toncini and colleagues’ (2018) recent paper a tool was developed based upon tracking and recording participants mouse movements whilst completing 76 questions deriving from numerous psychometric assessment instruments alongside questions regarding the experiment itself. 30 out of the 76 questions were categorised as simple questions and the remaining 46 questions were categorised as complex and thus required greater levels of cognitive load. Through the analysis of behavioural responses to these questions (by tracking mouse movements), this resulted in an impressive accuracy rate of 96% when identifying simulated malingers (Monaro, Toncini et al., 2018). Indeed, this is impressive and the practical implications of such an approach for clinical forensic practice are vast, although further research and development are required until such a tool could be implemented. The use of malingering detection approaches based upon identifying cognitive load as outlined above certainly demonstrates a move in the right direction for the detection of malingering and certainly overcomes some of the limitations associated with psychometric measures of malingering (Monaro, Gamberini et al., 2018) and clinicians’ assessments of malingering (Cartwright et al., 2019).

Due to the recent success of applying the cognitive load approach to malingering, this led the current researchers to question whether cognitive load or lack of cognitive load could be an influential predictor in an individual’s decision to malinger. As a result, the present study seeks to explore whether those with a more sophisticated knowledge of mental disorder may be more likely to malinger as the cognitive load required to lie would be reduced. In a study which analysed the British social attitudes survey data, Holman (2015) evidenced that, in general, the UK public has significantly more knowledge regarding depression as opposed to other disorders such as schizophrenia or asthma obtaining a diagnosis accuracy score of 72% in comparison to 35% and 62% respectively. Indeed, this suggests that the publics’ knowledge of depression is particularly pronounced, arguably due to the many campaigns aimed at increasing depression literacy in order to improve national mental health.

Accurate knowledge of mental disorder clearly can be positive, however, the present article aims to investigate whether such knowledge could be associated with negative behaviours such as malingering. The present paper investigates the following research question, does a participant’s level of knowledge regarding depression affect their attitude towards malingering the disorder? The experimental hypothesis of the present study is that higher levels of depression knowledge will be associated with malingering being perceived less seriously. This hypothesis will be explored measuring participants’ attitudes towards malingering depression in order to receive benefit payments due to being unemployed and the malingering of depression to receive compensation for work placed stress. Both contexts have been chosen due to the paucity of research exploring these two areas which are clearly vulnerable to those willing to malinger depression.

## Method

### Ethics

The present study was designed in accordance with the British Psychological Society’s code of human research ethics and was approved by Leeds Trinity University’s Ethics Committee.

### Participants

The sample consisted of 155 students studying at a UK university. As can be seen in Table 2 the sample comprised of 123 females and 32 males with a mean age of 23.5 (*SD* = 9.35). Students were recruited from advertisements sent via email to students on the following programmes: Criminology, Psychology, and Business Studies.

### Materials

#### Brief Depression Literacy Scale

After a search of the literature which resulted in two scales being created to assess depression literacy; Gabriel and Violato’s (2009) 27 item scale which achieved an internal reliability of α = .68, and the 22-item depression literacy questionnaire which achieved an internal reliability of α = .70 (Griffiths et al, 2004). For the present study, the two scales measured all aspects of depression literacy and thus were not necessary for investigating the present research question. The reason for this being is that the present study aims only to assess whether knowledge of depression influenced malingering as opposed to the wider constructs associated with depression literacy. Therefore, the Brief Depression Knowledge Scale was created comprising of 10 true or false questions which were generated based on the DSM-5 (APA, 2013) diagnostic criteria for depression. As discussed in the results section the internal reliability of the scale was good reaching KR(20) = .69. The scale comprised of the following true or false questions:

1. Feelings of sadness, emptiness, and hopelessness nearly every day is a symptom of depression
2. Diminished interest or pleasure in almost all activities is a symptom of depression
3. Inability to sleep or excessive sleep is a symptom of depression
4. Weight loss at a change of more than 5% a month is a symptom of depression
5. Weight gain at a change of more than 5% a month is a symptom of depression
6. Fatigue or loss of energy is a symptom of depression
7. Feelings of restlessness is a symptom of depression
8. Feelings of worthlessness or excessive or inappropriate guilt is a symptom of depression
9. Diminished ability to think or concentrate or indecisiveness is a symptom of depression
10. Recurrent thoughts of death or suicidal thoughts are a symptom of depression

In order to calculate a participant’s total depression knowledge score, participants were allocated one point for every question they answered as being true except for question 3 where they were allocated a point for answering the question as false. Following this, a total score can be calculated out of ten as a measure of a participant’s level of knowledge regarding depression.

#### Brief Social Desirability Scale

The Brief Social Desirability Scale (Haghighat, 2007) was used to capture the levels of social desirability due to the present article exploring contentious social behaviours. A discussion of social desirability is not provided here but should the reader wish to explore the theoretical underpinnings of social desirability, Tracey’s (2016) review is recommended. In short, social desirability is measured in the present study due to malingering being socially unacceptable and therefore participants may respond in a socially desirable way as opposed to honestly. The scale comprises of four questions and allows researchers to assess whether social desirability is a confounding variable. The four questions are as follows:

1. Do you always practice what you preach?
2. Do you always keep your promises no matter how inconvenient they may be?
3. Would you smile at people every time you meet them?
4. Would you ever lie to people?

### Procedure

Once participants had agreed to take part they were directed to an online survey portal (Bristol Online Surveys) which displayed the study information sheet. Upon reading the information sheet participants completed the study consent form before being asked several demographic questions. Following the demographic questions, participants were required to complete the Brief Depression Literacy Scale, followed by the Brief Social Desirability Scale (Haghighat, 2007).

Upon completion of these questions, participants were asked to read two vignettes which are displayed in Table 1, and were randomised to control for order effects.

**Table 1:** Vignette Questions Displayed to Participants

Malingering Strategy	Vignette and question descriptions	
Vignette	Vignette A: You have recently been going through a stressful period at work. Please answer the following questions in relation to how likely you would be to do the following.	Vignette B: You have recently been made unemployed and have made an application for benefit payments. Please answer the following questions in relation to how likely you would be to do the following.
Par- M	You are suffering from minor symptoms of depression following the stressful period at work, how likely are you to exaggerate these symptoms in order to claim a larger amount of personal injury compensation?	You are suffering from minor symptoms of depression due to being unemployed. How likely are you to exaggerate these symptoms in order to obtain increased benefits?
Pure- M	Following your stressful experience at work you are not suffering from any symptoms of depression. How likely would you be to state that you were to a medical professional in order to obtain financial compensation?	Following being made unemployed you are not suffering from any symptoms of depression. How likely would you be to state that you have been in order to obtain increased benefits?
F-Imp	You found yourself suffering from symptoms of depression before you experienced a stressful period at work, how likely are you to claim that you started suffering from these symptoms after the stressful work period in order to receive personal injury compensation?	You found yourself suffering from minor symptoms of depression before your period of unemployment. How likely are you to claim that you started suffering from these symptoms due to being unemployed in order to obtain increased benefits?

*Note.* Par-M = partial malingering; Pur-M = pure malingering; F-Imp = false imputation.

The first vignette placed participants in a situation whereby they had experienced stress due to work and then participants were asked three questions regarding how likely they would be to malinger depression. The three questions investigated how likely they would be to use an Par-M strategy, a Pure-M strategy, and an F-Imp strategy. Again, the order in which the three questions were displayed was randomised. Participants were required to respond to these questions using a 5 point Likert scale (not very likely, not likely, somewhat likely, likely, very likely). The second vignette placed participants in the same three questions as described above, however, the context changed to benefit payments. Following completion, participants were displayed with a debrief form.

### Analysis

Non-parametric statistics were utilised due to the assumption of normality being violated with the Shapiro-Wilk test revealing that all dependent variables did not have a normal distribution. The following non-parametric inferential statistics were used in the present study: Spearman’s rho, Mann-Whitney U test, Friedman’s repeated measures ANOVA, and the Wilcoxon signed rank test. Variables included in the analysis are as follows: participant demographics (including a self-report history of depression), measures of malingering, social desirability scores, and knowledge of depression scores.

## Results

Table 2 displays the demographic information of the participants included in the present study.

**Table 2:** Participants’ Demographic Information

Demographic variable	Statistic
**Age**	*M* = 23.50 (*SD* = 9.35)
Sex	
Females	79.4% (*n* = 123)
Males	20.6% (*n* = 32)
History of depressive symptomology	
No	47.1% (*n* = 73)
Yes	52.9% (*n* = 82)
History of claiming disability allowance	
No	93.5% (*n* = 145)
Yes	6.5% (*n* = 10)
History of claiming compensation	
No	89% (*n* = 138)
Yes	11% (*n* = 17)
**Depression literacy score**	*M* = 8.11 (*SD* = 1.90)
**Social desirability score**	*M* = 1.89 (*SD* = 1.06)

*Note. N* = 155.

To determine the extent to which socially desirable responding influenced the participants’ responses, Spearman’s rho correlations were undertaken. As can be seen in Table 3 Spearman’s rho correlations revealed that social desirability was significantly negatively correlated with four out of the six questions asked following the two vignettes. Thus, demonstrating that the results should be interpreted with caution as social desirability appears to be a confounding variable.

**Table 3:** Spearman’s Rho Correlations for the Malingering Strategies Across the Two Vignettes and Social Desirability Total Score

Malingering Strategy	*rs* for Social Desirability
Pure-M disability allowance	-.27**
Pure-M compensation at work	-.12
Par-M disability allowance	-.23**
Par-M compensation at work	-.28**
F-Imp disability allowance	-.35**
F-Imp compensation at work	-.14

*Note. N* = 155. Par-M = partial malingering; Pur-M = pure malingering; F-Imp = false imputation.

**p < .01.

This study primarily aimed to investigate whether depression knowledge affects the likelihood of participants’ taking part in a hypothetical malingering strategy, the Brief Depression Knowledge Scale was created. A Kuder-Richardson KR(20) analysis was run to determine the overall test reliability; the analysis indicated that the Brief Depression Literacy Scale had moderate reliability KR(20) = .69. Participants’ obtained an average score of *M* = 8.12 (*SD* = 1.90). Interpreting the KR(20) and the average score of 81% suggests that the depression knowledge questionnaire did not sufficiently discriminate those with high depression knowledge from those with low depression knowledge. Alternatively, it could be the case that participants within the present study had a very good knowledge of symptoms associated with depression. To determine whether depression knowledge affected the participants’ responses to the vignettes, further Spearman’s rho correlations were undertaken; no significant correlations emerged at the < .01 level (*p* = .37 - .87).

Participants were asked prior to taking part in the survey whether they have previously experienced symptoms of depression. To determine whether those participants who have previously experienced symptoms of depression are more likely to engage in malingering strategies Mann-Whitney U tests were undertaken and the results are displayed in Table 3.

As can be seen in Table 4 only one significant difference occurred. Those who had suffered from depressive symptoms in the past were more likely to malinger using an F-Imp strategy to claim compensation due to the stress experienced at work (*Mdn* = 2.00, *M* = 2.22) than those who have never experienced symptoms of depression (*Mdn* = 2.00 *M* = 1.89), *U* = 2854, *p* < .038, *r* = .17. Additionally, a further Man-Whitney U test was conducted to determine whether those who have experienced depressive symptoms in the past differ in their levels of depression knowledge than those who have not. The Man-Whitney U test indicated that those who have experienced depressive symptoms in the past (*Mdn* = 9.00, *M* = 8.48) scored significantly higher than those who had not (*Mdn* = 8.00, *M* = 7.71) on the depression literacy questionnaire *U* = 2379, *p* < .024, *r =* -.16.

**Table 4:** Likelihood of Malingering and Previous History of Depressive Symptomology

Malingering Vignette	History of depression (*n* = 82)		No history of depression (*n* = 73)		*U*	*p*	*r*
	*Mdn*	*M*	*Mdn*	*M*			
Pure-M disability allowance	1.00	1.29	1.00	1.42	2703	.19	-.11
Pure-M compensation at work	1.00	1.57	1.00	1.70	2860	.59	-.04
Par-M disability allowance	2.00	2.04	2.00	2.12	2918	.78	-.02
Par-M compensation at work	1.50	1.70	2.00	1.97	2650	.18	-.11
F-Imp disability allowance	2.00	2.04	2.00	2.12	2443	.60	-.04
F-Imp compensation at work	2.00	1.89	2.00	2.22	2854	< .038	-.17

*Note. N* = 155. Par-M = partial malingering; Pur-M = pure malingering; F-Imp = false imputation.

A further area of exploration was to determine whether the three malingering strategies were perceived with differing severity. To do this, Friedman’s repeated measures ANOVA was undertaken. Friedman’s ANOVA revealed a significant difference between the three malingering strategies in the vignettes examining compensation in the workplace χ^2^(2, *N* = 155) = 29.86, *p* < .001. Post-hoc pairwise comparisons using the Wilcoxon signed rank test indicated significant differences between: Par-M (*Mdn* = 2.00, *M =* 1.83) and Pure-M (*Mdn* = 1.00, *M* = 1.63), *Z* = -2.63, *p* < .009, *r* = .21; F-Imp (*Mdn* = 2.00, *M* = 2.05) and Par-M (*Mdn* = 2.00, *M* = 1.83), *Z* = -3.28, *p < .*001, *r* = .26; and F-Imp (*Mdn* = 2.00, *M* = 2.05) and Pure-M (*Mdn* = 1.00, *M* = 1.63) *Z* = -4.42, *p* < .001, *r* = .36. The Friedman’s ANOVA investigating whether there is a significant difference between the three malingering strategies for claiming disability allowance evidenced that the three strategies were not viewed differently χ^2^(2, *N* = 155) = 4.76, *p* = .09.

To determine whether the situation in which an individual malingers affects the likelihood of participants engaging in malingering Wilcoxon signed rank tests were undertaken. The first Wilcoxon test indicated that participants were significantly more likely to utilise a Pure-M (*Mdn* = 1.00 *M* = 1.63) for financial compensation in the workplace as opposed to using an Pure-M strategy to claim benefits (*Mdn* = 1.35, *M* = 1.00), *Z* = 3.68, *p* < .001, *r* = .30. Conversely, a second Wilcoxon test indicated that participants were significantly more likely to utilise a Par-M to claim benefits (*Mdn* = 2.00 *M* = 2.16) as opposed to using a Par-M strategy to claim financial compensation in the workplace (*Mdn* = 2.00, *M* = 1.83), *Z* = 4.17, *p* < .001, *r* = .33. The third Wilcoxon test indicated that the situation in which participants were asked about their likeliness of engaging in an F-Imp strategy did not result in a significant difference *Z* = .42, *p* = .67.

## Discussion

This article addressed an important and emerging area of research examining whether a relationship exists between depression knowledge and malingering. The findings reported here would suggest that an individual’s knowledge of mental disorder does not affect their likelihood of engaging in malingering for a financial reward and as a result the experimental hypothesis is not supported. Having said this, the Brief Knowledge of Depression scale used was unable to differentiate those with high levels of depression knowledge from those with lower levels. Indeed, it could be the case that the sample included in the present study have particularly high levels of knowledge or it might be the case that the UK population, in general, are well educated regarding depression. At the very least, this study corroborates previous research demonstrating that as a society we seem to have a high level of knowledge when it comes to depression (Holman, 2015). As a result, future research is needed to provide a better understanding of the relationship between knowledge of depression and malingering.

The present study provides evidence that malingering is not a singular construct. The three strategies of malingering were perceived differently, with Pure-M being viewed more severely, followed by Par-M, and then F-Imp. However, this was only true for the workplace vignette as participants did not differentiate between the three malingering strategies in the benefit vignette. As a result, it is argued that the context in which an individual malingers is a significant factor in the causation of the behaviour. Interestingly, when the two contexts were compared individuals were more inclined to commit the more serious form of malingering (Pure-M) to obtain financial compensation in the workplace, but were more likely to use a Par-M strategy to obtain benefit payments than to behave in the same way for financial compensation in the workplace. This finding is complicated but provides further evidence of the complexity of the relationship between the context where one might malinger and an individual’s choice to malinger. Individuals perceive totally fabricating symptoms to be significantly more serious if it is for benefit payments and it is suggested here, that this is perhaps due to a stigma attached to those who cheat the benefits system. A converse relationship is found when individuals are asked about simply exaggerating symptoms of depression and doing this to attain benefits is viewed more favourably than workplace compensation. However, F-Imp was viewed as the most favourable in both vignettes but the context in which participants were asked about this behaviour did not affect their responses.

A further important finding is that those participants who self-reported previous histories of depression were significantly more likely to state that they would be willing to use an F-Imp strategy to claim workplace compensation, however, the same relationship was not found for the benefit vignette. To use an F-Imp strategy, participants need to have a previous history of mental disorder but considering that this study was simply exploring participants’ perceptions provides evidence that it is this type of malingering that is most likely to occur with individuals who have a history of mental disorder. This is concerning considering that it is argued to be the most difficult to spot (Cartwright et al., 2016; Hall & Hall, 2006; Resnick & Knoll, 2005).

The present article provides further support for Rogers (1990) adaptational model of malingering by demonstrating how contexts can alter an individual’s attitude towards malingering. Furthermore, the present article suggests that cultural social forces may play a role in the decision to malinger whilst providing further evidence for the role of previous mental disorder. As a result, this research provides an indication of some factors that underpin Rogers (1990) adaptational model, however, an individual’s knowledge of the mental disorder is not one of these. Having said this, it is imperative that future research investigates this hypothesis utilising a sophisticated measure of depression knowledge. Future research would be encouraged to measure all aspects of depression literacy and not simply individuals’ knowledge of depressive symptomology. The exploration of different mental disorders should also be encouraged within future research, as it may be the case that society, in general, has high levels of knowledge regarding depression.

It must be acknowledged that this article is a theoretical approach to the study of malingering exploring attitudes and intentions as opposed to actual behaviour. Critics of this method will rightly outline that what people say and what they actually do are often two different things. Indeed, this is correct but the study of malingering is particularly problematic and alternative methodologies also suffer from internal reliability issues due to the problem of obtaining the ground truth. Furthermore, the reader must acknowledge that the sample size of the present paper is small and not representative of the UK demographic, however, it is argued that it is sufficient to investigate this study’s exploratory hypothesis of whether depression literacy influences malingering using bivariate analyses to detect correlation coefficients of 0.3 with an alpha level of 0.05 and a power of 80% (Bujang & Baharum, 2016). Future research nonetheless would be encouraged to increase the sample size to detect even smaller changes in the correlation coefficient, whilst utilising a cross-sectional design to recruit a representative demographic.

In summary, the present paper offers a unique contribution to the malingering literature through the exploration of a previously untested hypothesis aimed at enhancing the understanding of malingering for financial compensation. The current understanding of malingering which outlines that it is context specific and the result of a cost-benefit analysis, is in no way sufficient or helpful to forensic examiners. As a result, the present paper provides a methodology for developing the theoretical explanations of malingering through testing the underlying constructs that may be influential during the would-be malingerer’s cost-benefit analysis.
